# Prevalence of Neuroradiological Abnormalities in First-Episode Psychosis: A Systematic Review and Meta-analysis

**DOI:** 10.1001/jamapsychiatry.2023.2225

**Published:** 2023-07-12

**Authors:** Graham Blackman, Giulia Neri, Omar Al-Doori, Maria Teixeira-Dias, Asif Mazumder, Thomas A. Pollak, Emily J. Hird, Nikolaos Koutsouleris, Vaughan Bell, Matthew J. Kempton, Philip McGuire

**Affiliations:** 1 Department of Psychiatry, University of Oxford, Warneford Hospital, Oxford, United Kingdom; 2 Department of Psychosis Studies, Institute of Psychiatry, Psychology and Neuroscience, King’s College London, London, United Kingdom; 3 South London and Maudsley NHS Foundation Trust, London, United Kingdom; 4 Department of Neuroradiology, King’s College Hospital NHS Foundation Trust, London, United Kingdom; 5 Department of Radiology, Guy’s and St Thomas’ NHS Foundation Trust, London, United Kingdom; 6 Institute of Cognitive Neuroscience, University College London, London, United Kingdom; 7 Department of Psychiatry and Psychotherapy, Ludwig-Maximilian-University, Munich, Germany; 8 Max-Planck Institute of Psychiatry, Munich, Germany; 9 Research Department of Clinical, Educational and Health Psychology, University College London, London, United Kingdom

## Abstract

**Importance:**

Individuals presenting with first-episode psychosis (FEP) may have a secondary (“organic”) etiology to their symptoms that can be identified using neuroimaging. Because failure to detect such cases at an early stage can have serious clinical consequences, it has been suggested that brain magnetic resonance imaging (MRI) should be mandatory for all patients presenting with FEP. However, this remains a controversial issue, partly because the prevalence of clinically relevant MRI abnormalities in this group is unclear.

**Objective:**

To derive a meta-analytic estimate of the prevalence of clinically relevant neuroradiological abnormalities in FEP.

**Data Sources:**

Electronic databases Ovid, MEDLINE, PubMed, Embase, PsychINFO, and Global Health were searched up to July 2021. References and citations of included articles and review articles were also searched.

**Study Selection:**

Magnetic resonance imaging studies of patients with FEP were included if they reported the frequency of intracranial radiological abnormalities.

**Data Extraction and Synthesis:**

Independent extraction was undertaken by 3 researchers and a random-effects meta-analysis of pooled proportions was calculated. Moderators were tested using subgroup and meta-regression analyses. Heterogeneity was evaluated using the *I^2^* index. The robustness of results was evaluated using sensitivity analyses. Publication bias was assessed using funnel plots and Egger tests.

**Main Outcomes and Measures:**

Proportion of patients with a clinically relevant radiological abnormality (defined as a change in clinical management or diagnosis); number of patients needed to scan to detect 1 such abnormality (number needed to assess [NNA]).

**Results:**

Twelve independent studies (13 samples) comprising 1613 patients with FEP were included. Of these patients, 26.4% (95% CI, 16.3%-37.9%; NNA of 4) had an intracranial radiological abnormality, and 5.9% (95% CI, 3.2%-9.0%) had a clinically relevant abnormality, yielding an NNA of 18. There were high degrees of heterogeneity among the studies for these outcomes, 95% to 73%, respectively. The most common type of clinically relevant finding was white matter abnormalities, with a prevalence of 0.9% (95% CI, 0%-2.8%), followed by cysts, with a prevalence of 0.5% (95% CI, 0%-1.4%).

**Conclusions and Relevance:**

This systematic review and meta-analysis found that 5.9% of patients presenting with a first episode of psychosis had a clinically relevant finding on MRI. Because the consequences of not detecting these abnormalities can be serious, these findings support the use of MRI as part of the initial clinical assessment of all patients with FEP.

## Key Points

**Question:**

How common are neuroradiological abnormalities in first-episode psychosis?

**Findings:**

In this systematic review and meta-analysis, we found approximately 6% of patients with first-episode psychosis had an abnormality that required a change in clinical management. The number of patients needed to scan to detect 1 clinically relevant abnormality was estimated to be 18.

**Meaning:**

These findings support the routine use of magnetic resonance imaging as part of the initial assessment in patients presenting with first-episode psychosis.

## Teaser

This systematic review and meta-analysis examines data from studies of patients with first-episode psychosis to estimate the prevalence of clinically relevant neuroradiological abnormalities detected using magnetic resonance imaging.

## Introduction

The early diagnosis of secondary psychosis, where a psychotic disorder is caused by another medical condition, is an indispensable but complex diagnostic task in psychiatry. Because several causes of secondary psychosis result in structural brain abnormalities,^1^ brain imaging is considered essential by many psychiatrists during the assessment phase.^2^ Magnetic resonance imaging (MRI) is a safe and well-tolerated^3^ technique that has high sensitivity for detecting intracranial abnormalities. Since its introduction more than 40 years ago, structural MRI has become increasingly available, and the costs of scanning have progressively reduced.^4^ However, there is no consensus as to whether MRI scanning should be a mandatory part of the clinical assessment of patients presenting with a first episode of psychosis (FEP). Some guidelines recommend scanning all patients with FEP,^5^ while others recommend that MRI be restricted to cases in which a secondary cause is suspected.^6^

Most radiological abnormalities in patients with FEP are incidental and do not require any clinical action. However, a minority of abnormalities lead to a change to a patient’s clinical care. A barrier to resolving the debate about the routine use of MRI in the assessment of FEP has been uncertainty about the prevalence of clinically relevant abnormalities, with estimates ranging from 0%^7^ to more than 10%.^8^ Beyond guidelines for individual clinical management, there is also the issue of population health. In otherwise healthy individuals, white matter hyperintensities reliably predict later cognitive decline, greater cerebrovascular risk, and increased mortality in epidemiological studies,^9,10^ suggesting that the presence of neuroradiological abnormalities may be an indicator of neurological health.

We sought to clarify the prevalence of intracranial abnormalities in FEP by undertaking the first meta-analysis of such studies to our knowledge. We also sought to establish the prevalence of clinically relevant abnormalities that led to a change in diagnosis or management. In addition, we examined the influence of study, patient, and imaging characteristics on outcome.

## Methods

A systematic review and meta-analysis was conducted in accordance with Meta-analysis of Observational Studies in Epidemiology (MOOSE)^11^ and Preferred Reporting Items for Systematic Reviews and Meta-analyses (PRISMA) guidelines,^12^ and the study was prospectively registered on PROSPERO (CRD42020140917). See the eMethods in Supplement 1 for details of the search strategy, eligibility criteria, and data extraction and encoding. In brief, we searched the databases Ovid, MEDLINE, PubMed, Embase, PsychINFO, and Global Health up to July 2021. References and citations of included articles and review articles were also searched.

### Quality Assessment

Included studies were assessed for the risk of bias using a 10-item tool developed for prevalence studies^13^ (eTable 3 in Supplement 1). The sum was calculated to derive a summary score. Studies were categorized based on the overall score as high (0-3), moderate (4-6), or low (7-10) risk of bias using well-established cutoffs. Studies at high risk of bias were excluded from the meta-analysis.

### Outcomes

A radiological abnormality was defined as any intracranial finding, regardless of the evidence to suggest a causal relationship with psychosis. Radiological abnormalities were further categorized by clinical relevance. A clinically relevant finding was defined as an abnormality that was reported by the study authors as having led to a change in management (eg, referral to a medical specialty) or diagnosis. Abnormalities were also grouped into the following neuroanatomical categories: white matter, vascular (excluding white matter), ventricular, cyst, pituitary, tumor, cerebral atrophy, and other (ie, not falling into any of the aforementioned categories) by a psychiatrist (G.B.) and a neuroradiologist (A.M.), with the latter blind to diagnosis (eMethods in Supplement 1).

### Statistical Analysis

For each study, the proportion of patients with FEP and a radiological abnormality was calculated, along with the 95% CI based on the Score (Wilson) method.^14^ A Freeman-Tukey double arcsine transformation^15^ was used to stabilize the variance because the proportion of abnormal scans was expected to be low.^16^ Transformed proportions were meta-analyzed using a random-effects inverse variance method^17^ as methodological heterogeneity was anticipated. To assess the clinical usefulness of MRI, the estimated number of patients needed to be scanned to detect 1 abnormality (number needed to assess [NNA]) was calculated, based on the reciprocal of the prevalence estimate,^18^ which is comparable with the numbers-needed-to-treat^19^ statistic: NNA = 1 / (proportion with abnormality).

We also estimated the prevalence of the neuroanatomical subtypes of abnormalities (eTable 4 in Supplement 1). In addition, for studies that included a healthy control group, we calculated the risk ratio (relative risk) to explore the specific association between neuroradiological abnormalities and psychosis.

The significance level was set to *P* ≤ .05, and all analyses were performed using R version 4.2.1^20^ with meta-analyses performed using meta.^21,22^ Further details on the statistical analysis are reported in the eMethods in Supplement 1, and all code and data are included in an online archive (link available on request).

### Assessment of Heterogeneity

Heterogeneity was assessed using the Cochran *Q* statistic, as well as the *I^2^* index, which is independent of the number of studies. Risk of publication bias was assessed thorough visual inspection of funnel plots and an Egger test.^23^ Modifiers of clinically relevant abnormalities were assessed through subgroup analysis and meta regression provided there were sufficient data points. For categorical variables, we explored the effect of the sample (research vs clinical) and field strength (3 T vs <3 T) using subgroup analysis based on the Cochran *Q* statistic. For continuous variables, we explored the effect of sample age, psychosis duration, and year of publication using meta-regression provided at least 6 studies could be included.^24^

### Sensitivity Analyses

Sensitivity analyses were performed to determine the effect of studies (1) with a mean patient age older than 35 years, (2) where assessment was performed by a nonradiologist, and (3) based on a research sample. Influential study analysis using the leave-1-out paradigm^25^ was performed using the dmetar package.^26^ This was performed to identify any study with an excessive influence on the pooled effect size and/or that contributed substantially to between-study heterogeneity.

## Results

### Search Results and Study Selection

The search strategy yielded 1682 publications from the database search and other sources. After duplicates were removed and abstracts screened, 240 publications were reviewed in full. eFigure 1 in Supplement 1 shows the PRISMA flowchart. In 1 study, patients with FEP had been pooled with patients with multiepisode psychosis^8^; however, it was possible to estimate the proportion of abnormalities in the FEP subgroup group based on published details and consultation with the study authors (eMethods in Supplement 1). In another study, only white matter abnormalities were reported,^27^ so this study was not entered into the main meta-analysis.

### Study Characteristics

Twelve studies were eligible,^3,7,8,27,28,29,30,31,32,33,34,35^ with no overlapping samples (eTable 1 in Supplement 1 contains study characteristics). Studies were published between 1991 and 2021 and reported a pooled sample of 1613 patients with FEP. Nine studies reported clinically relevant abnormalities,^3,7,8,27,28,29,30,32,34^ with a pooled sample of 1318 patients. Eight studies included a healthy control group,^3,8,27,29,30,33,34,35,36^ with a pooled sample of 3265 patients (FEP = 1399; control = 1866). (eTable 2 in Supplement 1 describes the recruitment, screening, and matching of healthy controls for each study.) Studies were conducted in Europe (n = 7), North America (n = 4), Australasia (n = 1), and South America (n = 1). Ten studies excluded patients in whom a potential secondary cause of psychosis was suspected before neuroimaging, such as a positive finding on a neurological examination (not reported in 2 studies).

In a minority of studies, the total number of abnormalities in the sample were reported, rather than the number of patients with an abnormality. A post hoc sensitivity analysis was therefore performed to restrict to studies that reported the total number of patients with an abnormality. All studies reporting clinically relevant abnormalities reported this at the patient level.

### Participant Characteristics

The FEP sample size ranged from 20^35^ to 349 patients.^8^ Mean age ranged from 20 to 60 years, and the proportion of female patients ranged from 27% to 70%. Five studies reported data from routine clinical practice, and 6 studies reported data from clinical research studies. One study reported data from both routine clinical practice and clinical research.^3^ For the purposes of subsequent analysis, this study was split into research and clinical subsamples (therefore, 13 samples are considered henceforth). Antipsychotic status at the time of neuroimaging was reported in 6 samples (n = 714). Among these, the proportion of patients receiving antipsychotic medication was 65%. Duration of psychosis was reported in 6 samples (n = 665) and ranged between 4 and 52 weeks with the exception of 1 study, which had a mean duration of 90 weeks.^35^

### Neuroimaging Characteristics

Scanner field strength was reported in 10 samples, with 1.5 T (n = 6) being the most common. MRI scans were interpreted by a neuroradiologist in 9 samples. In the other 3 samples, MRI scans were reported by a general radiologist (n = 1) or a psychiatrist (n = 1), or the clinician was unspecified (n = 1). In 6 samples, raters were blind to clinical status (unreported in 8 samples).

### Quality Assessment and Risk of Bias

The quality assessment score ranged from 4 to 8 of 10 (eTable 2 in Supplement 1). Overall, 10 samples were at medium risk and 3 were at low risk of bias. No studies were at high risk of bias.

### Prevalence of Radiological Abnormalities

**Figure 1.**
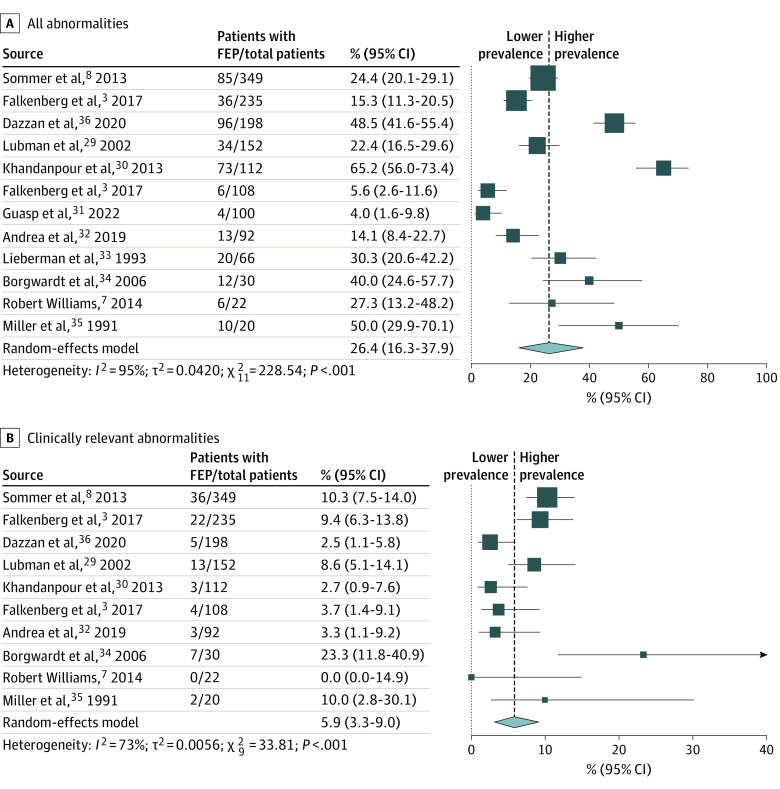
Forest Plots of Magnetic Resonance Imaging Abnormalities in First-Episode Psychosis (FEP) The size of each box is proportional to the weight of the study in relation to the pooled estimate.

The proportion of patients with any abnormality was calculable for 12 samples (11 studies) because 1 study only reported the presence or absence of white matter abnormalities.^27^ The pooled prevalence was 26.4% (95% CI, 16.3%-37.9%), with a corresponding NNA of 4 (95% CI, 3-7) (Figure 1). The *I^2^* statistic was 95%, indicating a high degree of heterogeneity. The proportion of patients with a clinically relevant abnormality was calculable for 10 samples (9 studies). In the other samples, clinically relevant abnormalities were grouped with non–clinically relevant abnormalities.^29,31,33^ The pooled prevalence was 5.9% (95% CI, 3.2%-9.0%), with a corresponding NNA of 18 (95% CI, 12-31). The *I^2^* statistic was 73%, indicating moderate heterogeneity.

### Prevalence of Radiological Abnormalities by Anatomical Type

**Figure 2.**
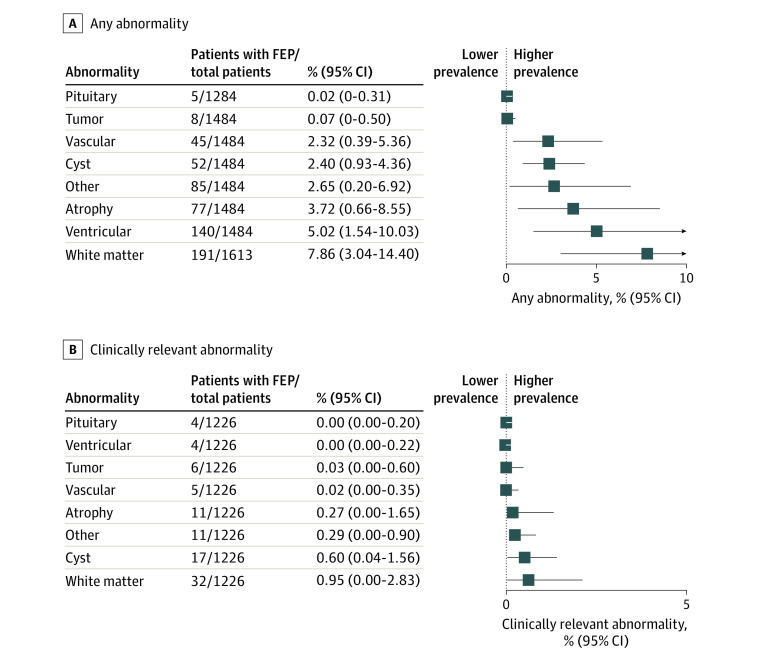
Forest Plots of Magnetic Resonance Imaging Abnormalities in First-Episode Psychosis (FEP) Grouped by Neuroanatomical Type

As part of the secondary analysis, we calculated the prevalence of specific neuroanatomical abnormalities among patients with FEP (Figure 2). Overall, white matter abnormalities were the most common (typically white matter hyperintensities), with a prevalence of 7.9% (95% CI, 3.0% to 14.4%), followed by ventricular abnormalities (typically ventricular enlargement), with a prevalence of 5.0% (95% CI, −1.5% to 10.0%) (eFigure 3 in Supplement 1). Among clinically relevant abnormalities, white matter abnormalities were the most common, with a prevalence of 0.9% (95% CI, 0% to 2.8%), followed by cysts, with a prevalence of 0.5% (95% CI, 0% to 1.4%) (Figure 2 and eFigure 4 in Supplement 1).

### Relative Risk of Radiological Abnormalities

We also calculated the pooled risk ratio of neuroanatomical abnormalities in patients with FEP vs healthy controls. Patients with FEP had a relative risk of 2.8 (95% CI, 1.3-5.9; k = 9 studies) for any radiological abnormality compared with heathy controls (eFigure 5A in Supplement 1). Among abnormalities that were clinically relevant, patients with FEP had a relative risk of 1.5 (95% CI, 0.8-2.8) compared with heathy controls (eFigure 5B in Supplement 1); however, a leave-1-out sensitivity analysis (below) indicated that this may be an underestimate.

### Influence of Potential Effect Modifiers on Prevalence

Meta-regression found no association between the prevalence of clinically relevant abnormalities and publication year (k = 10, *P* = .07) or sample age (k = 9, *P* = .95). There were insufficient samples (k = 3) to explore the effect of psychosis duration. Subgroup analysis found no association for the effect of sample type (k = 10, *P* = .99) or field strength (k = 12, *P* = .16).

### Sensitivity Analyses

We repeated the analysis excluding samples (1) with a mean patient age older than 35 years, (2) where assessment was performed by a nonradiologist, and (3) that recruited patients for research. Removing studies under any 1 of these conditions did not result in pooled estimates becoming nonsignificant. Leave-1-out sensitivity analysis did not identify any influential samples for the meta-analysis of prevalence (eFigure 6 in Supplement 1). Leave-1-out sensitivity analysis indicated that the study by Khandanpour et al^30^ was influential in the meta-analysis of relative risk for any abnormality, and removal adjusted the pooled relative risk to 1.8 (95% CI, 1.1-3.2). For the meta-analysis of relative risk for clinically relevant abnormalities, the study by Sommer et al^8^ was identified as influential, and removal adjusted the pooled relative risk to 2.1 (95% CI, 1.1-4.0).

### Publication Bias

Inspection of funnel plots suggested no clear evidence of publication bias (eFigure 2 in Supplement 1), which was confirmed by nonsignificant Egger test results for studies reporting any abnormalities (*P* = .36) and those reporting clinically relevant abnormalities (*P* = .70).

## Discussion

The estimated prevalence of a radiological MRI abnormality in patients with FEP was 26%, while that of a clinically relevant abnormality was 6%. Patients with FEP had a significantly higher prevalence of radiological abnormalities overall, as well as clinically relevant abnormalities compared with healthy controls, after removal of an outlier. White matter abnormalities, predominantly small hyperintensities, were the most common finding overall and the most common clinically relevant finding. The NNA to detect 1 clinically relevant abnormality was 18.

Although the prevalence of neuroradiological abnormalities in FEP has previously been explored in systematic reviews,^4,37,38^ to our knowledge, this is the first study to derive a meta-analytic estimate based on MRI data. Previous studies have reported conflicting results. The largest MRI study of patients with psychosis (n = 656) reported a higher prevalence of clinically relevant abnormalities (10.3%) in their first episode subsample compared with our meta-analytic estimate but essentially found no difference from healthy controls, who had a similarly high prevalence of clinically relevant abnormalities (11.8%).^8^ This study is notable for being the only one in our meta-analysis that reported the prevalence of clinically relevant abnormalities to be lower in patients with psychosis than in controls and was identified as an outlier in the leave-1-out sensitivity analysis. Studies exploring radiological abnormalities in patients with psychosis using computed tomography (CT) have yielded substantially lower estimates than MRI.^4,39^ This likely reflects the relative insensitivity of CT to detect intracranial abnormalities in patients with psychosis.

In otherwise healthy individuals, the prevalence of incidental clinically relevant brain abnormalities found on MRI is estimated to be 1.4%^40^ to 2.7%.^18^ In our study, we were able to derive the first meta-analytic estimate of the relative risk of clinically relevant brain abnormalities in FEP compared with asymptomatic healthy individuals. Our findings suggest a 2-fold increased risk, once adjusted for outliers. Research MRI studies have identified widespread differences in gray and white matter density in FEP compared with controls. However, these studies typically use voxel-based morphometry and involve alterations that are too small to be detected by the naked eye. Although most radiological abnormalities in FEP do not necessitate a change in management, it is worth noting that these apparently benign findings may be associated with relatively poor outcomes across the life span^41^ and a marker of neurovascular health.^42^ This suggests that they could reflect the macroscopic sequelae of suboptimal brain development and as such may represent determinants of a poor outcome, even if they do not lead to a diagnosis of secondary psychosis.

The most common neuroradiological abnormality was white matter abnormalities, predominantly small hyperintensities. They were also the most common clinically relevant abnormality reported. This finding is consistent with independent neuroimaging evidence that psychosis is associated with widely distributed anatomical and functional dysconnectivity.^43,44,45^ White matter lesions are closely associated with neuroinflammatory processes in psychosis,^46^ as well as immune-mediated neurological disorders such as multiple sclerosis,^47^ supporting an etiological role of the immune system in psychosis.

Interestingly, we found the prevalence of brain tumors in FEP was very low (with the estimated NNA to detect 1 tumor of around 1000) despite this being one of the main concerns of psychiatrists. However, because all the studies in this meta-analysis excluded patients with clinical evidence suggestive of a secondary medical (“organic”) cause, our results are likely to underestimate the true prevalence of tumors in patients with FEP more broadly, as such cases are more likely to present with neurologic features, such as apraxia, visual field deficits, and anomia.^48^

The heterogeneity between studies in the proportion of patients with any type of abnormality was large. In contrast, heterogeneity for clinically relevant abnormalities was moderate. Between-study differences in design, eligibility criteria, neuroimaging methods, and radiological assessment may have contributed to this statistical heterogeneity. We explored its basis using subgroup analysis and meta-regression. The former found no difference between studies based on sample type, rater, or field strength, and the latter found that the effects of patient age and publication year were not significant. We were not able to explore the effect of psychosis duration because of insufficient data.

We assessed the robustness of the findings using sensitivity analyses. One study^49^ was identified as an outlier in the meta-analysis of risk for clinical abnormalities, and its removal resulted in the risk ratio becoming significant. Furthermore, the results remained robust to several sensitivity analyses. Our group-level estimates assumed that each patient had a maximum of 1 type of abnormality, and findings did not change substantially at a group level when we excluded studies in which this assumption could not be confirmed.

Should MRI be routinely performed in patients with FEP? One approach to resolving this debate is to consider the net clinical benefit. We were able to ascertain that 1 in 18 patients had a change in management after an MRI, and therefore it could be argued they received some clinical benefit. In contrast, clinical risks associated with MRI scanning are minimal, and most patients find the procedure acceptable.^3^ Another approach is to consider the economic implications. The financial costs of a brain MRI vary considerably, and therefore the economic case for routine screening is also likely to vary. In Europe, the average cost is around $264 (€250), including evaluation by a radiologist. Based on the estimated NNA, the cost to detect 1 clinically relevant abnormality is approximately $4752 (€4500). In comparison, the financial cost is substantially higher in the United States. However, the potential costs associated with failing to identify a clinically relevant abnormality (that may include a potentially reversible cause) are also likely to be higher. While further analysis is indicated to explore the net economic benefits, provisional evaluation based on clinical grounds would favor offering MRI to all patients with FEP.

### Strengths and Limitations

This meta-analysis provides the most precise estimate of the prevalence of neuroradiological abnormalities in FEP in the literature to date. Subgroup and meta-regression permitted the exploration of moderating factors and causes of heterogeneity, such as study characteristics and imaging parameters. Furthermore, by comparing neuroradiological abnormalities in FEP with healthy controls, we were able to determine the specificity of these abnormalities. Importantly, in most studies, FEP samples were matched with healthy controls. Other strengths included a rigorous approach to study identification and data extraction. Furthermore, because the meta-analysis focused on patients with FEP, the findings are unlikely to have been confounded by the influence of chronic illness or its treatment.

This study also had limitations. First, the studies we examined may not have included patients who were particularly unwell and/or lacked capacity. Second, around half of the studies involved patients who had undergone MRI as part of research rather than routine clinical care, and all the studies had excluded patients in whom there was clinical evidence of a potential secondary cause (based on examination and/or psychiatric assessment). These factors are likely to have resulted in an underestimate of the prevalence of clinically relevant radiological abnormalities in FEP, suggesting the true figure may be higher. Third, we assumed each patient had only 1 type of radiological abnormality. However, in a few studies, this could not be confirmed, which may have inflated the overall estimate (of note, this limitation did not apply to our estimate of clinically relevant abnormalities). Fourth, because we used aggregate data, we were unable to explore the influence of potentially relevant patient-level characteristics. Fifth, information on duration of illness and antipsychotic exposure was unavailable in several studies. Finally, included studies mostly consisted of relatively small samples, which reduces statistical precision.

### Future Research

Follow-up data would help determine the proportion of clinically relevant radiological abnormalities that are treatable. Similarly, it would be useful to clarify whether the presence of radiological abnormalities are associated with adverse long-term clinical outcomes. If this was the case, this may suggest a role for MRI in providing prognostic information in addition to its diagnostic role. Secondary causes of psychosis are associated with particular clinical variables, such as visual hallucinations^50,51,52^ and delusions of misidentification.^53^ Systematic assessment of these risk factors could complement the use of MRI to help clinicians identify patients with a secondary etiology. Further research is also indicated to explore the optimal MRI parameters for detecting radiological abnormalities.

## Conclusions

This systematic review and meta-analysis found that around 6% of patients presenting with psychosis have a clinically relevant radiological abnormality on MRI, with a corresponding NNA of 18. These findings provide a rationale for the use of MRI in the clinical assessment of all patients presenting with psychosis. As the availability of MRI increases and its costs decrease, it is becoming increasingly difficult to justify not making MRI a mandatory part of the clinical assessment of FEP.

## Notes

Supplement 1.
eMethods
**eFigure 1.** PRISMA flow diagram **eFigure 2.** Prevalence of MRI abnormalities in FEP: funnel plot of studies **eFigure 3.** Prevalence of MRI abnormalities in FEP: forest plots of MRI abnormalities by anatomical subtype **eFigure 4.** Prevalence of MRI abnormalities in FEP: forest plots of clinically relevant MRI abnormalities by anatomical subtype **eFigure 5.** Risk ratio of MRI abnormalities in FEP: forest plots of all MRI abnormalities in psychosis **eFigure 6.** Forest plot of clinically relevant MRI abnormalities: leave one out sensitivity analysis **eTable 1.** Summary of included studies **eTable 2.** Summary of recruitment, screening, and matching criteria of healthy controls **eTable 3.** Newcastle Ottawa Scale results **eTable 4.** Neuroanatomical Groupings
eReferences

Supplement 2. Data sharing statement
