# Supplementary material for: Prevalence of Neuroradiological Abnormalities in First-Episode Psychosis: A Systematic Review and Meta-analysis
Source: JAMA Psychiatry. 2023 Jul 12;80(10):1047–54. doi: 10.1001/jamapsychiatry.2023.2225 (PMC10339221; doi:10.1001/jamapsychiatry.2023.2225)
Supplement: Supplement 1. — eMethods eFigure 1. PRISMA flow diagram eFigure 2. Prevalence of MRI abnormalities in FEP: funnel plot of studies eFigure 3. Prevalence of MRI abnormalities in FEP: forest plots of MRI abnormalities by anatomical subtype eFigure 4. Prevalence of MRI abnormalities in FEP: forest plots of clinically relevant MRI abnormalities by anatomical subtype eFigure 5. Risk ratio of MRI abnormalities in FEP: forest plots of all MRI abnormalities in psychosis eFigure 6. Forest plot of clinically relevant MRI abnormalities: leave one out sensitivity analysis eTable 1. Summary of included studies eTable 2. Summary of recruitment, screening, and matching criteria of healthy controls eTable 3. Newcastle Ottawa Scale results eTable 4. Neuroanatomical Groupings eReferences [file jamapsychiatry-e232225-s001.pdf]

Supplemental Online Content

Blackman G, Neri G, Al-Doori O, et al. Prevalence of neuroradiological abnormalities in first-episode psychosis: a systematic review and meta-analysis. *JAMA Psychiatry*. Published online July 12, 2023. doi:10.1001/jamapsychiatry.2023.2225

eMethods
eFigure 1. PRISMA flow diagram
eFigure 2. Prevalence of MRI abnormalities in FEP: funnel plot of studies
eFigure 3. Prevalence of MRI abnormalities in FEP: forest plots of MRI abnormalities by anatomical subtype
eFigure 4. Prevalence of MRI abnormalities in FEP: forest plots of clinically relevant MRI abnormalities by anatomical subtype
eFigure 5. Risk ratio of MRI abnormalities in FEP: forest plots of all MRI abnormalities in psychosis
eFigure 6. Forest plot of clinically relevant MRI abnormalities: leave one out sensitivity analysis
eTable 1. Summary of included studies
eTable 2. Summary of recruitment, screening, and matching criteria of healthy controls
eTable 3. Newcastle Ottawa Scale results
eTable 4. Neuroanatomical Groupings
eReferences

This supplemental material has been provided by the authors to give readers additional information about their work.

© 2023 Blackman G et al. *JAMA Psychiatry.*

eMethods

Search strategy

Pairs of researchers (G.N., O.A., M.D) rated articles for inclusion. Articles that were approved by both were included and discrepancies were resolved by an independent ratter (GB). A three-step approach was taken in the search. First, the electronic databases Ovid, MEDLINE, PubMed, Embase, PsychINFO, and Global Health were searched from inception up to July 2021. The following search terms were used: “first episode psychosis” OR “psychosis” AND “magnetic resonance imaging” OR “MRI” OR “sMRI” OR “structural magnetic resonance imaging” AND “structural brain abnormalities” OR “brain abnormalities” OR “neuroradiological abnormalities.” Second, the references of eligible articles were manually reviewed. Third, the references of systematic reviews on structural neuroimaging findings in psychosis were assessed (Albon et al., 2008; Forbes et al., 2019; Goulet et al., 2009). Articles were screened based on the title and abstract before the full article was reviewed to confirm eligibility.

Eligibility criteria

Inclusion criteria were a FEP patient group (as defined by the study authors) and the frequency of intracranial abnormalities (as identified through a radiological assessment of structural MRI). Exclusion criteria were insufficient data to permit calculation of prevalence estimates; studies that were restricted to pre-specified neuroanatomical regions (e.g., midline); and studies that included patients with clinical signs or symptoms suggestive of a neurological disorder (e.g. seizures). Studies were included irrespective of whether they assessed all intracranial abnormalities, or specific types (e.g., white matter abnormalities), however studies falling into the latter category were not incorporated into the main meta-analysis. Where more than one study used the same or overlapping samples, the study with the larger sample was included. Any setting or study design was considered, excluding case reports. There were no age or language restrictions.

Data extraction and encoding

Data extraction was independently performed by three researchers using a piloted standardized form, with a fourth researcher resolving any discrepancies. For each study, information on the study characteristics, sample, imaging parameters and intracranial abnormalities was also extracted.

Estimation of the proportion of abnormalities

For one study (Sommer et al., 2013), only the total frequency and proportion of all patients with psychosis, including first episode and multi episode psychosis were reported. The total number of patients with a first episode of psychosis (n=349), and multi episode psychosis (n=307) were reported and differences in frequency were not significant (p=0.45). Based on this information, we were able to model frequencies of abnormalities within the first episode of psychosis, and multi episode psychosis that would generated the reported pooled frequency. We assumed first episode psychosis was less strongly associated with MRI abnormalities than multiple episode psychosis. We ran simulations of different prevalence in each group that resulted in a pooled prevalence in keeping with the study results. For each simulation, we ran a chi square test. We then chose the combination that most closely approximaetd the P value reported in the manuscript. We repeated this for clinically relevant abnormalities. For the sub-types of abnormalities, we assumed that there was no significant difference between first episode and multi-episode subgroups. We then adjusted the frequency of sub-types of abnormalities in proportion of the entire sample who were first episode psychosis.

© 2023 Blackman G et al. *JAMA Psychiatry.*

PRISMA (2009) CHECKLIST

Section/Topic	#	Checklist Item	Reported on page #
TITLE			
Title	1	Identify the report as a systematic review, meta-analysis, or both.	1
ABSTRACT			
Structured summary	2	Provide a structured summary including, as applicable: background; objectives; data sources; study eligibility criteria, participants, and interventions; study appraisal and synthesis methods; results; limitations; conclusions and implications of key findings; systematic review registration number.	2
INTRODUCTION			
Rationale	3	Describe the rationale for the review in the context of what is already known.	4
Objective	4	Provide an explicit statement of questions being addressed with reference to participants, interventions, comparisons, outcomes, and study design (PICOS).	4
METHODS			
Protocol and registration	5	Indicate if a review protocol exists, if and where it can be accessed (e.g., Web address), and, if available, provide registration information including registration number.	5
Eligibility criteria	6	Specify study characteristics (e.g., PICOS, length of follow-up) and report characteristics (e.g., years considered, language, publication status) used as criteria for eligibility, giving rationale.	5
Information sources	7	Describe all information sources (e.g., databases with dates of coverage, contact with study authors to identify additional studies) in the search and date last searched.	5
Search	8	Present full electronic search strategy for at least one database, including any limits used, such that it could be repeated.	5
Study selection	9	State the process for selecting studies (i.e., screening, eligibility, included in systematic review, and, if applicable, included in the meta-analysis).	5
Data collection process	10	Describe method of data extraction from reports (e.g., piloted forms, independently, in duplicate) and any processes for obtaining and confirming data from investigators.	5 , eMethods
Data items	11	List and define all variables for which data were sought (e.g., PICOS, funding sources) and any assumptions and simplifications made.	6
Risk of bias in individual studies	12	Describe methods used for assessing risk of bias of individual studies (including specification of whether this was done at the study or outcome level), and how this information is to be used in any data synthesis.	5, 3Table 3
Summary measures	13	State the principal summary measures (e.g., risk ratio, difference in means).	6
Synthesis of results	14	Describe the methods of handling data and combining results of studies, if done, including measures of consistency (e.g., I^2) for each meta-analysis.	6

© 2023 Blackman G et al. *JAMA Psychiatry.*

Risk of bias across studies	15	Specify any assessment of risk of bias that may affect the cumulative evidence (e.g., publication bias, selective reporting within studies).	6-7
Additional analyses	16	Describe methods of additional analyses (e.g., sensitivity or subgroup analyses, meta-regression), if done, indicating which were pre-specified.	7
RESULTS			
Study selection	17	Give numbers of studies screened, assessed for eligibility, and included in the review, with reasons for exclusions at each stage, ideally with a flow diagram.	8
Study characteristics	18	For each study, present characteristics for which data were extracted (e.g., study size, PICOS, follow-up period) and provide the citations.	8
Risk of bias within studies	19	Present data on risk of bias of each study and, if available, any outcome level assessment (see item 12).	9, sTable 1
Results of individual studies	20	For all outcomes considered (benefits or harms), present, for each study: (a) simple summary data for each intervention group (b) effect estimates and confidence intervals, ideally with a forest plot.	10
Synthesis of results	21	Present results of each meta-analysis done, including confidence intervals and measures of consistency.	9
Risk of bias across studies	22	Present results of any assessment of risk of bias across studies (see Item 15).	12
Additional analysis	23	Give results of additional analyses, if done (e.g., sensitivity or subgroup analyses, meta-regression [see Item 16]).	11-12
DISCUSSION			
Summary of evidence	24	Summarize the main findings including the strength of evidence for each main outcome; consider their relevance to key groups (e.g., healthcare providers, users, and policy makers).	13
Limitations	25	Discuss limitations at study and outcome level (e.g., risk of bias), and at review-level (e.g., incomplete retrieval of identified research, reporting bias).	16
Conclusions	26	Provide a general interpretation of the results in the context of other evidence, and implications for future research.	16
FUNDING			
Funding	27	Describe sources of funding for the systematic review and other support (e.g., supply of data); role of funders for the systematic review.	1

© 2023 Blackman G et al. *JAMA Psychiatry.*

MOOSE CHECKLIST

Criteria	Brief description of how the criteria were handled in the meta-analysis
Reporting of background should include	
Problem definition	Individuals presenting with first episode psychosis may have an "organic" aetiology identifiable using neuroimaging, and failure to detect these early can have serious consequences for the patient. However, the proportion of patients that have MRI abnormalities that are clinically significant remains unclear.
Hypothesis statement	FEP patients are at increased odds of having structural abnormalities, as compared to healthy controls.
Description of study outcomes	Neuroradiological abnormality
Type of exposure or intervention used	n/a
Type of study designs used	prevalence
Study Population	Patients with first episode psychosis
Reporting of search strategy should include	
Qualifications of searchers	Academic qualifications defined on title page
Search strategy, including time period included in the synthesis and key words	Full selection procedures (data bases, time period, and search terms) in Methods, search strategy
Databases and registries searched	Ovid MEDLINE, PubMed, Embase, PsychINFO, and Global Health databases<br>databases
Search Software used, name and version	Ovid: ovidsp.ovid.com/<br>PubMed: www.ncbi.nlm.nih.gov/pubmed/
Use of hand searching	references of includes articles
List of citations located and those excluded, including justifications	Defined in Results
Method of addressing articles published in languages other than English	Only studies in English were considered
Methods of handling abstracts and unpublished studies	Defined in Methods
Reporting of methods should include	
Description of relevance or appropriateness of studies assembled for assessing the hypothesis to be tested	Described in Results, Study Selection. The minimum dataset to be included in the meta-analysis was the number of FEP with a neuro-radiological abnormality
Rationale for the selection and coding of data	Based on review of the literature and discussion with experts in neuroradiology
Assessment of confounding	Sensitivity analyses performed assessing impact of skewed data and environmental/physiological confounds
Assessment of study quality	Assessed using the Newcastle Ottawa Scale
Assessment of heterogeneity	Assessed using I^2
Description of statistical methods in sufficient detail to be replicated	Details provided in methods section and supplementary information. References cited.

© 2023 Blackman G et al. *JAMA Psychiatry.*

Provision of appropriate tables and graphics	Study identification described using PRISMA diagram (supplementary information), forest plots for all analyses provided. Raw data of included provided in supplementary information.
Reporting of results should include	
Graph summarising individual study estimates and overall estimate	Details provide in Results using forest plots for all analyses provided, including the pooled prevalence of radiological abnormalities and abnormalities by anatomical subtype.
Table giving descriptive information for each study included	Supplemental table 1
Results of sensitivity testing	Described in Results (sensitivity analyses section)
Indication of statistical uncertainty of findings	Results report 95% confidence interval and associated probability (p) value.
Reporting of discussion should include	
Quantitative assessment of bias	Approach to assessment of bias reported in Methods. Additional provided in supplementary materials.
Justification for Exclusion	Justification for excluding studies at high risk of bias in sensitivity analysis reported in Methods
Assessment of quality of included studies	Study quality reported in Results.
Reporting of conclusions should involve	
Considerations of alternative explanations for observed results	In discussion alternative explanations for observed results described
Generalisation of the conclusions	Discussion outlines generalisability of findings to routine clinical practice
Guidelines for future research	Discussion details recommendations for future research
Disclosure of funding source	Defined on title page

© 2023 Blackman G et al. *JAMA Psychiatry.*

eFigure 1: PRISMA flow diagram

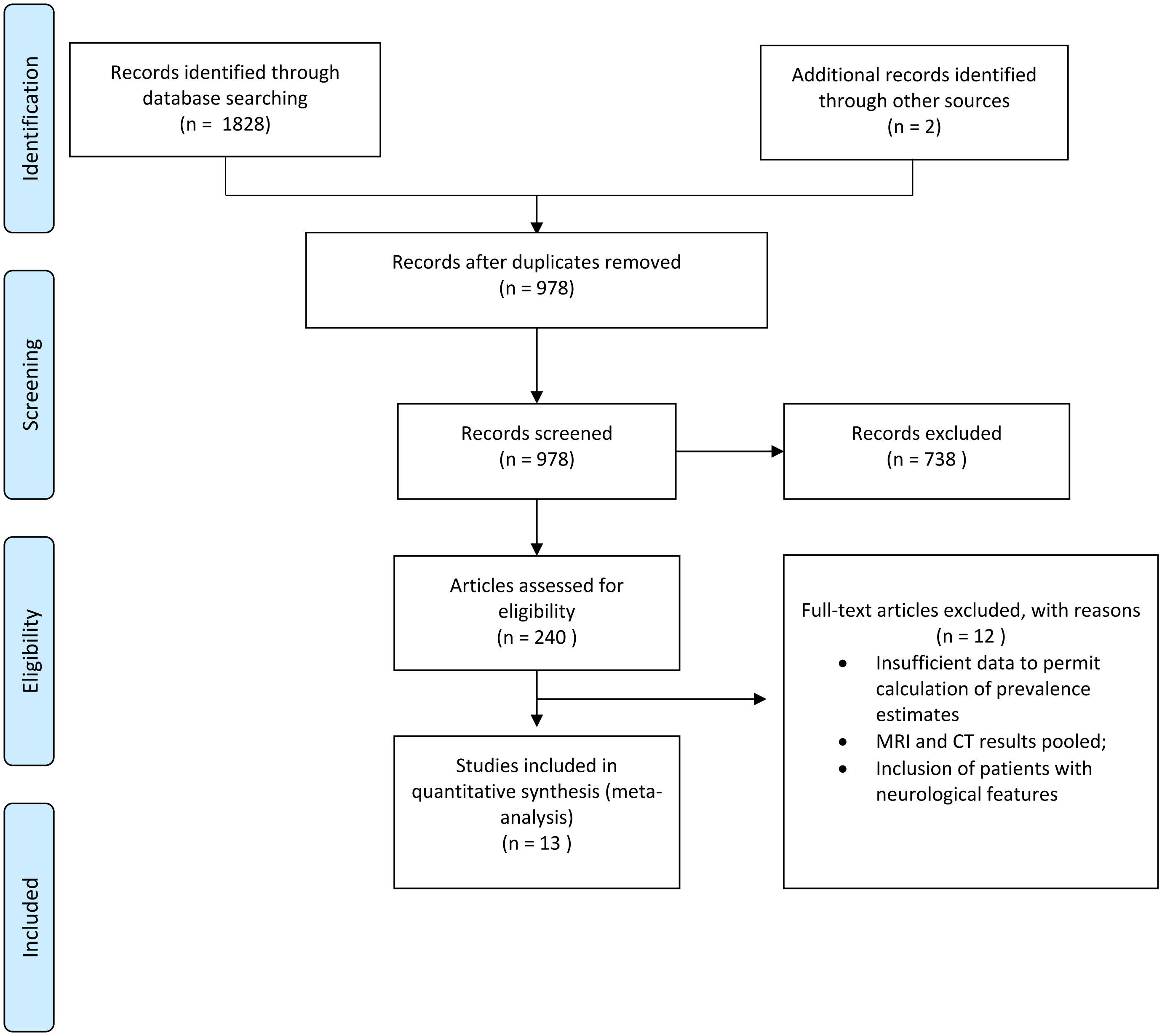

© 2023 Blackman G et al. *JAMA Psychiatry.*

eFigures section 2: Prevalence of MRI abnormalities in FEP: Funnel plot of studies

eFigure 2a: Funnel plot of studies – all abnormalities

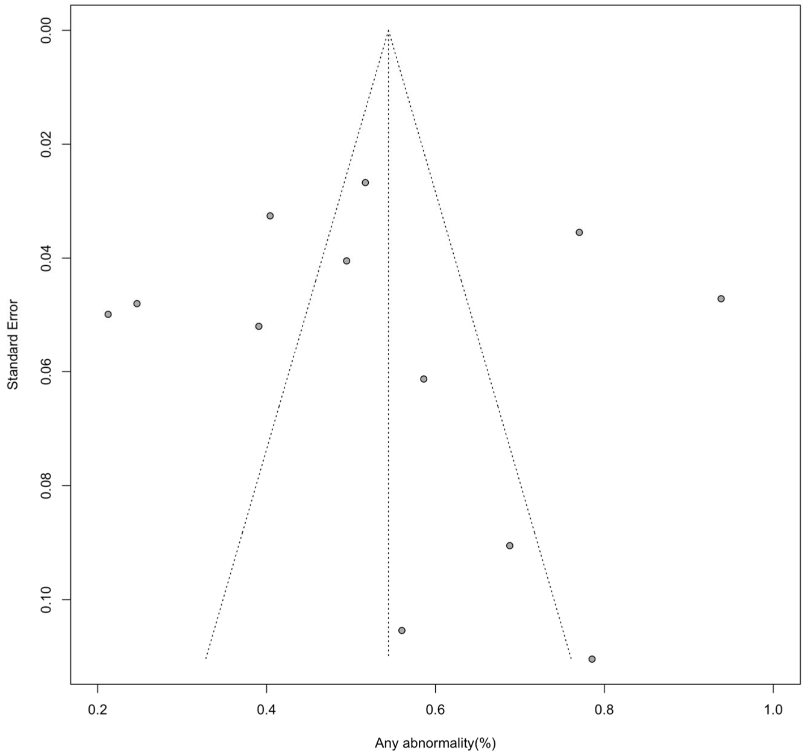

eFigure 2b: Funnel plot of studies – clinically relevant abnormalities

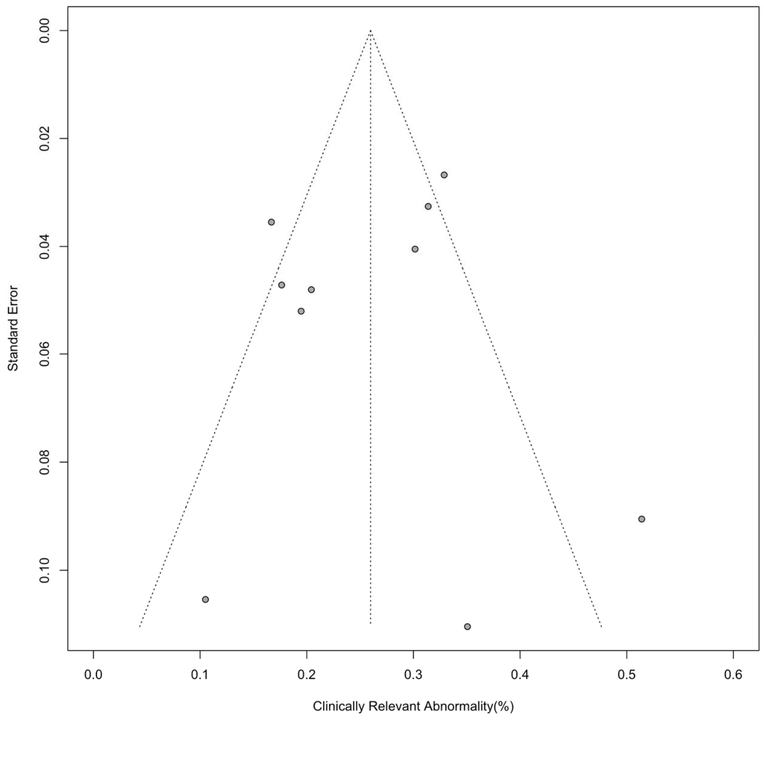

© 2023 Blackman G et al. *JAMA Psychiatry.*

eFigures Section 3: Prevalence of MRI abnormalities in FEP: Forest plots of MRI abnormalities by anatomical subtype

eFigure 3a - Forest plot - abnormality subtype: white matter

Study	Cases	Total	Prev(%)	95% C.I.
Lieberman 1993	0	66	0.0	[0.0; 6]
Miller 1991	0	20	0.0	[0.0; 16]
Falkenberg (r) 2017	1	108	0.9	[0.2; 5]
Guasp 2021	1	100	1.0	[0.2; 5]
Lubman 2002	8	152	5.3	[2.7; 10]
Falkenberg (c) 2017	14	235	6.0	[3.6; 10]
Williams 2014	1	22	4.5	[0.8; 22]
Borgwardt 2006	2	30	6.7	[1.8; 21]
Andrea 2019	7	92	7.6	[3.7; 15]
Sommer 2013	29	349	8.3	[5.8; 12]
Dazzan 2020	47	198	23.7	[18.3; 30]
Zanetti 2008	32	129	24.8	[18.2; 33]
Khandanpour 2012	49	112	43.8	[34.9; 53]
**Random effects model**		**.**	**7.9**	**[3.0; 14]**

Heterogeneity: $I^2 = 94\%$, $\tau^2 = 0.0310$, $\chi^2_{12} = 189.44$ ($p < 0.01$)

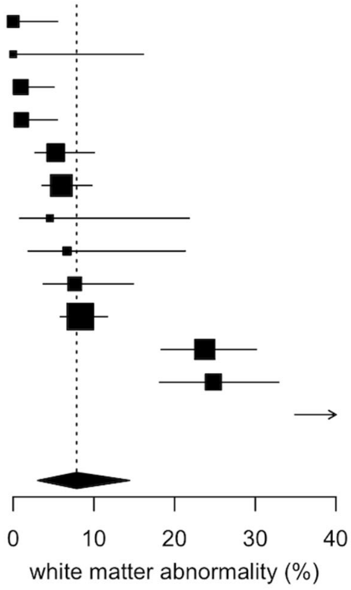

eFigure 3b - Forest plot - abnormality subtype: vascular abnormality

Study	Cases	Total	Prev(%)	95% C.I.
Falkenberg (r) 2017	0	108	0.0	[0.0; 3]
Lieberman 1993	0	66	0.0	[0.0; 6]
Falkenberg (c) 2017	1	235	0.4	[0.1; 2]
Borgwardt 2006	0	30	0.0	[0.0; 11]
Dazzan 2020	2	198	1.0	[0.3; 4]
Guasp 2021	1	100	1.0	[0.2; 5]
Andrea 2019	1	92	1.1	[0.2; 6]
Lubman 2002	2	152	1.3	[0.4; 5]
Sommer 2013	7	349	2.0	[1.0; 4]
Williams 2014	2	22	9.1	[2.5; 28]
Khandanpour 2012	23	112	20.5	[14.1; 29]
Miller 1991	6	20	30.0	[14.5; 52]
**Random effects model**		**.**	**2.3**	**[0.4; 5]**

Heterogeneity: $I^2 = 86\%$, $\tau^2 = 0.0133$, $\chi^2_{11} = 79.88$ ($p < 0.01$)

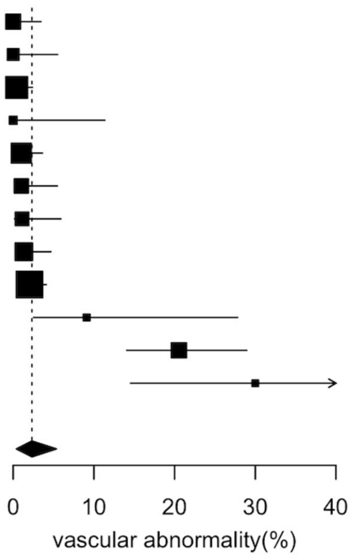

© 2023 Blackman G et al. *JAMA Psychiatry.*

eFigure 3c - Forest plot - abnormality subtype: cyst

Study	Cases	Total	Prev(%)	95% C.I.
Guasp 2021	0	100	0.0	[0.0; 4]
Lieberman 1993	0	66	0.0	[0.0; 6]
Miller 1991	0	20	0.0	[0.0; 16]
Khandanpour 2012	1	112	0.9	[0.2; 5]
Falkenberg (r) 2017	2	108	1.9	[0.5; 6]
Falkenberg (c) 2017	6	235	2.6	[1.2; 6]
Lubman 2002	4	152	2.6	[1.0; 7]
Sommer 2013	11	349	3.2	[1.8; 6]
Andrea 2019	4	92	4.3	[1.7; 11]
Williams 2014	1	22	4.5	[0.8; 22]
Dazzan 2020	19	198	9.6	[6.2; 14]
Borgwardt 2006	4	30	13.3	[5.3; 30]
**Random effects model**		**.**	**2.4**	**[0.9; 4]**

Heterogeneity: $I^2 = 69\%$, $\tau^2 = 0.0047$, $\chi^2_{11} = 35.22$ ($p < 0.01$)

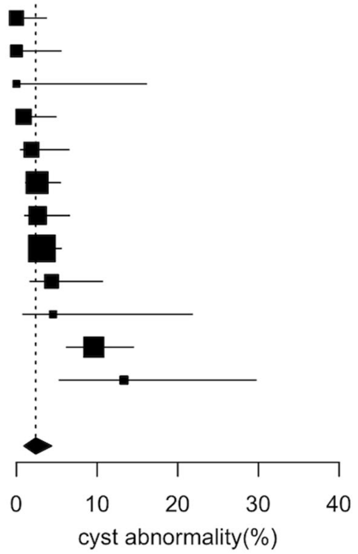

eFigure 3d - Forest plot - abnormality subtype: tumour

Study	Cases	Total	Prev(%)	95% C.I.
Falkenberg (c) 2017	0	235	0.0	[0.0; 1.6]
Lubman 2002	0	152	0.0	[0.0; 2.5]
Falkenberg (r) 2017	0	108	0.0	[0.0; 3.4]
Guasp 2021	0	100	0.0	[0.0; 3.7]
Andrea 2019	0	92	0.0	[0.0; 4.0]
Lieberman 1993	0	66	0.0	[0.0; 5.5]
Sommer 2013	1	349	0.3	[0.1; 1.6]
Williams 2014	0	22	0.0	[0.0; 14.9]
Dazzan 2020	3	198	1.5	[0.5; 4.4]
Khandanpour 2012	2	112	1.8	[0.5; 6.3]
Borgwardt 2006	1	30	3.3	[0.6; 16.7]
Miller 1991	1	20	5.0	[0.9; 23.6]
**Random effects model**		**.**	**0.1**	**[0.0; 0.5]**

Heterogeneity: $I^2 = 22\%$, $\tau^2 = 0.0006$, $\chi^2_{11} = 14.08$ ($p = 0.23$)

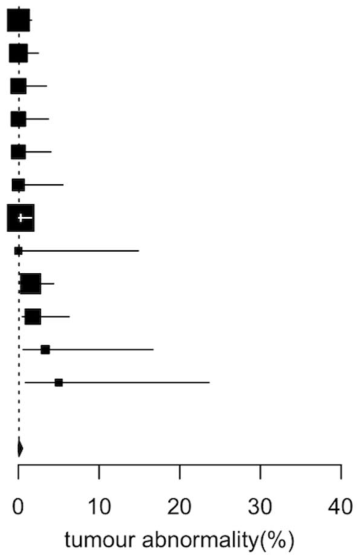

© 2023 Blackman G et al. *JAMA Psychiatry.*

eFigure 3e - Forest plot - abnormality subtype: atrophy

Study	Cases	Total	Prev(%)	95% C.I.
Falkenberg (c) 2017	0	235	0.0	[0.0; 2]
Falkenberg (r) 2017	0	108	0.0	[0.0; 3]
Andrea 2019	0	92	0.0	[0.0; 4]
Lieberman 1993	0	66	0.0	[0.0; 6]
Guasp 2021	1	100	1.0	[0.2; 5]
Lubman 2002	4	152	2.6	[1.0; 7]
Dazzan 2020	7	198	3.5	[1.7; 7]
Sommer 2013	19	349	5.4	[3.5; 8]
Williams 2014	2	22	9.1	[2.5; 28]
Miller 1991	2	20	10.0	[2.8; 30]
Borgwardt 2006	5	30	16.7	[7.3; 34]
Khandanpour 2012	37	112	33.0	[25.0; 42]
**Random effects model**		.	**3.7**	**[0.7; 9]**

Heterogeneity: $I^2 = 92\%$, $\tau^2 = 0.0251$, $\chi^2_{11} = 141.09$ ($p < 0.01$)

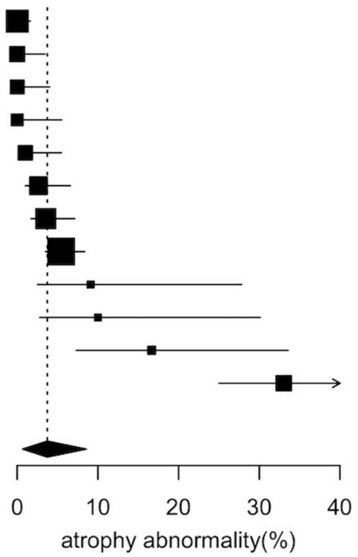

eFigure 3f - Forest plot - abnormality subtype: ventricular

Study	Cases	Total	Prev(%)	95% C.I.
Guasp 2021	0	100	0.0	[0.0; 4]
Williams 2014	0	22	0.0	[0.0; 15]
Miller 1991	0	20	0.0	[0.0; 16]
Khandanpour 2012	1	112	0.9	[0.2; 5]
Andrea 2019	1	92	1.1	[0.2; 6]
Falkenberg (r) 2017	2	108	1.9	[0.5; 6]
Borgwardt 2006	1	30	3.3	[0.6; 17]
Falkenberg (c) 2017	14	235	6.0	[3.6; 10]
Lubman 2002	10	152	6.6	[3.6; 12]
Sommer 2013	51	349	14.6	[11.3; 19]
Dazzan 2020	42	198	21.2	[16.1; 27]
Lieberman 1993	18	66	27.3	[18.0; 39]
**Random effects model**		.	**5.0**	**[1.5; 10]**

Heterogeneity: $I^2 = 91\%$, $\tau^2 = 0.0222$, $\chi^2_{11} = 125.88$ ($p < 0.01$)

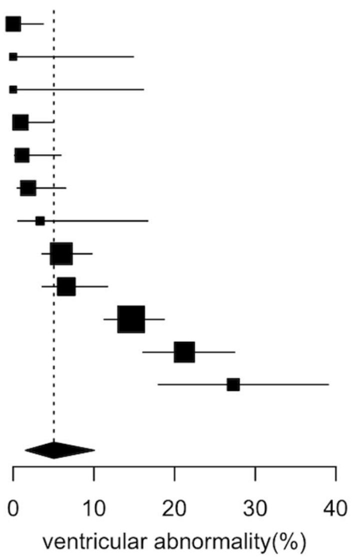

© 2023 Blackman G et al. *JAMA Psychiatry.*

eFigure 3g - Forest plot - abnormality subtype: pituitary

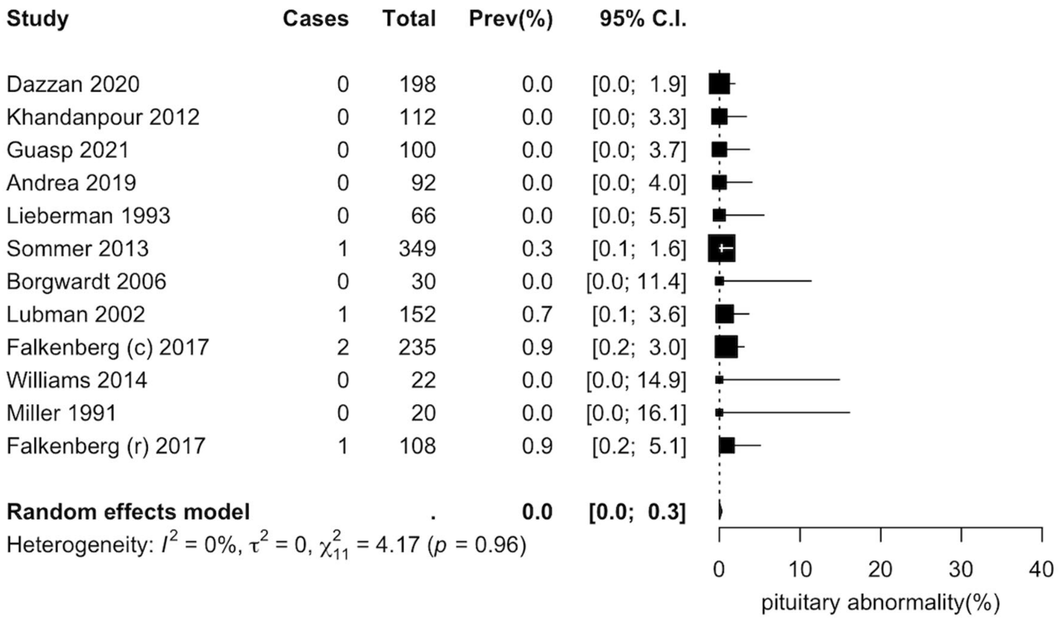

eFigure 3h - Forest plot - abnormality subtype: other

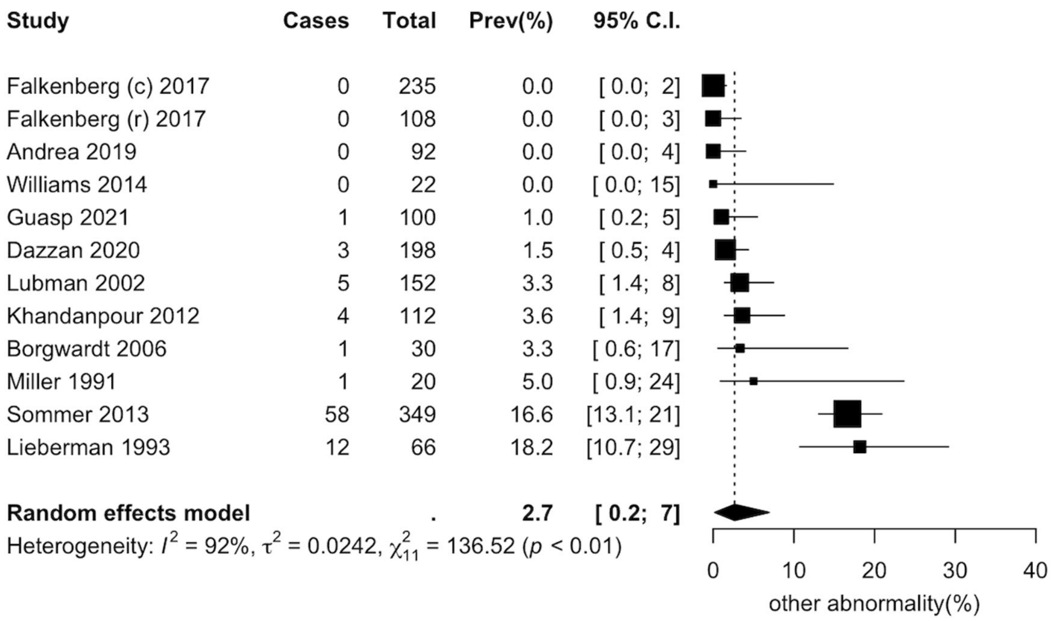

© 2023 Blackman G et al. *JAMA Psychiatry.*

eFigures Section 4: Prevalence of MRI abnormalities in FEP: Forest plots of clinically relevant MRI abnormalities by anatomical subtype

eFigure 4a - Forest plot - clinically relevant abnormality subtype: white matter

Study	Cases	Total	Prev(%)	95% C.I.
Dazzan 2020	0	198	0.0	[0.0; 2]
Zanetti 2008	0	129	0.0	[0.0; 3]
Khandanpour 2012	0	112	0.0	[0.0; 3]
Guasp 2021	0	100	0.0	[0.0; 4]
Borgwardt 2006	0	30	0.0	[0.0; 11]
Williams 2014	0	22	0.0	[0.0; 15]
Miller 1991	0	20	0.0	[0.0; 16]
Falkenberg (r) 2017	1	108	0.9	[0.2; 5]
Lubman 2002	3	152	2.0	[0.7; 6]
Sommer 2013	14	349	4.0	[2.4; 7]
Falkenberg (c) 2017	14	235	6.0	[3.6; 10]
Andrea 2019	.	92		
Lieberman 1993	.	66		
**Random effects model**		**.**	**0.6**	**[0.0; 2]**

Heterogeneity: $I^2 = 74\%$, $\tau^2 = 0.0057$, $\chi^2_{10} = 38.65$ ($p < 0.01$)

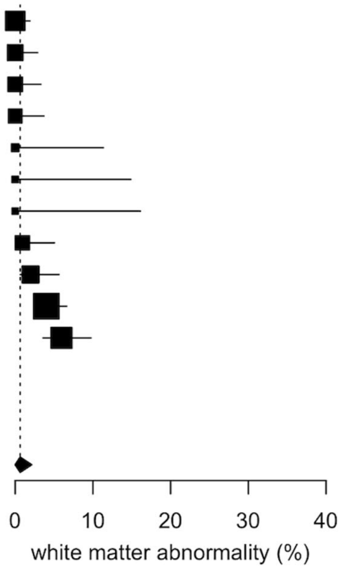

eFigure 4b - Forest plot - clinically relevant abnormality subtype: vascular

Study	Cases	Total	Prev(%)	95% C.I.
Dazzan 2020	0	198	0.0	[0.0; 1.9]
Khandanpour 2012	0	112	0.0	[0.0; 3.3]
Falkenberg (r) 2017	0	108	0.0	[0.0; 3.4]
Guasp 2021	0	100	0.0	[0.0; 3.7]
Falkenberg (c) 2017	1	235	0.4	[0.1; 2.4]
Sommer 2013	2	349	0.6	[0.2; 2.1]
Borgwardt 2006	0	30	0.0	[0.0; 11.4]
Williams 2014	0	22	0.0	[0.0; 14.9]
Miller 1991	0	20	0.0	[0.0; 16.1]
Lubman 2002	2	152	1.3	[0.4; 4.7]
Andrea 2019	.	92		
Lieberman 1993	.	66		
**Random effects model**		**.**	**0.0**	**[0.0; 0.3]**

Heterogeneity: $I^2 = 0\%$, $\tau^2 = 0$, $\chi^2_9 = 4.17$ ($p = 0.90$)

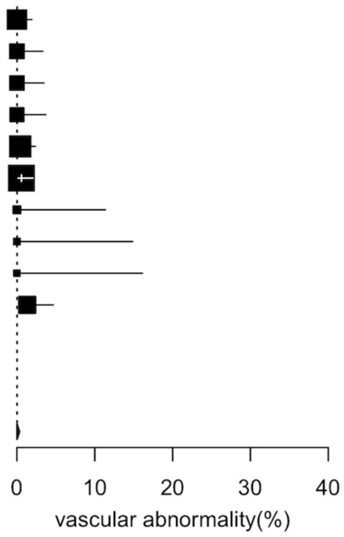

© 2023 Blackman G et al. *JAMA Psychiatry.*

eFigure 4c - Forest plot - clinically relevant abnormality subtype: cyst

Study	Cases	Total	Prev(%)	95% C.I.
Dazzan 2020	0	198	0.0	[0.0; 2]
Khandanpour 2012	0	112	0.0	[0.0; 3]
Guasp 2021	0	100	0.0	[0.0; 4]
Borgwardt 2006	0	30	0.0	[0.0; 11]
Williams 2014	0	22	0.0	[0.0; 15]
Miller 1991	0	20	0.0	[0.0; 16]
Sommer 2013	5	349	1.4	[0.6; 3]
Falkenberg (r) 2017	2	108	1.9	[0.5; 6]
Falkenberg (c) 2017	6	235	2.6	[1.2; 6]
Lubman 2002	4	152	2.6	[1.0; 7]
Andrea 2019	.	92		
Lieberman 1993	.	66		
**Random effects model**		**.**	**0.5**	**[0.0; 1]**

Heterogeneity: $I^2 = 36\%$, $\tau^2 = 0.0011$, $\chi^2_9 = 14.09$ ($p = 0.12$)

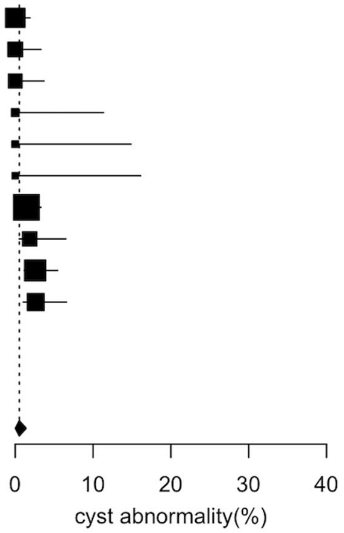

eFigure 4d - Forest plot - clinically relevant abnormality subtype: tumour

Study	Cases	Total	Prev(%)	95% C.I.
Sommer 2013	0	349	0.0	[0.0; 1.1]
Falkenberg (c) 2017	0	235	0.0	[0.0; 1.6]
Lubman 2002	0	152	0.0	[0.0; 2.5]
Falkenberg (r) 2017	0	108	0.0	[0.0; 3.4]
Guasp 2021	0	100	0.0	[0.0; 3.7]
Williams 2014	0	22	0.0	[0.0; 14.9]
Miller 1991	0	20	0.0	[0.0; 16.1]
Dazzan 2020	3	198	1.5	[0.5; 4.4]
Khandanpour 2012	2	112	1.8	[0.5; 6.3]
Borgwardt 2006	1	30	3.3	[0.6; 16.7]
Andrea 2019	.	92		
Lieberman 1993	.	66		
**Random effects model**		**.**	**0.0**	**[0.0; 0.5]**

Heterogeneity: $I^2 = 34\%$, $\tau^2 = 0.0010$, $\chi^2_9 = 13.59$ ($p = 0.14$)

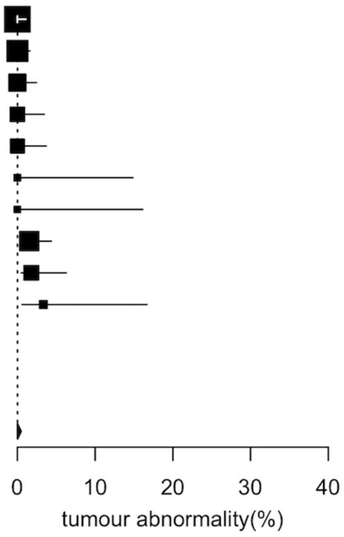

© 2023 Blackman G et al. *JAMA Psychiatry.*

eFigure 4e - Forest plot - clinically relevant abnormality subtype: atrophy

Study	Cases	Total	Prev(%)	95% C.I.
Falkenberg (c) 2017	0	235	0.0	[0.0; 2]
Dazzan 2020	0	198	0.0	[0.0; 2]
Lubman 2002	0	152	0.0	[0.0; 2]
Khandanpour 2012	0	112	0.0	[0.0; 3]
Falkenberg (r) 2017	0	108	0.0	[0.0; 3]
Guasp 2021	0	100	0.0	[0.0; 4]
Williams 2014	0	22	0.0	[0.0; 15]
Sommer 2013	4	349	1.1	[0.4; 3]
Miller 1991	2	20	10.0	[2.8; 30]
Borgwardt 2006	5	30	16.7	[7.3; 34]
Andrea 2019	.	92		
Lieberman 1993	.	66		
**Random effects model**		**.**	**0.2**	**[0.0; 1]**

Heterogeneity: I^2 = 69%, τ^2 = 0.0044, χ^2_9 = 28.72 (p < 0.01)

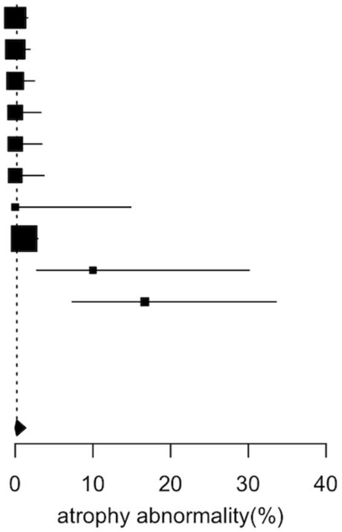

eFigure 4f - Forest plot - clinically relevant abnormality subtype: ventricular

Study	Cases	Total	Prev(%)	95% C.I.
Falkenberg (c) 2017	0	235	0.0	[0.0; 1.6]
Dazzan 2020	0	198	0.0	[0.0; 1.9]
Khandanpour 2012	0	112	0.0	[0.0; 3.3]
Falkenberg (r) 2017	0	108	0.0	[0.0; 3.4]
Guasp 2021	0	100	0.0	[0.0; 3.7]
Borgwardt 2006	0	30	0.0	[0.0; 11.4]
Lubman 2002	1	152	0.7	[0.1; 3.6]
Sommer 2013	3	349	0.9	[0.3; 2.5]
Williams 2014	0	22	0.0	[0.0; 14.9]
Miller 1991	0	20	0.0	[0.0; 16.1]
Andrea 2019	.	92		
Lieberman 1993	.	66		
**Random effects model**		**.**	**0.0**	**[0.0; 0.2]**

Heterogeneity: I^2 = 0%, τ^2 = 0, χ^2_9 = 4.79 (p = 0.85)

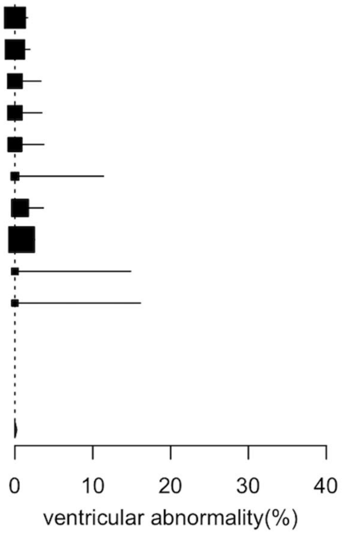

© 2023 Blackman G et al. *JAMA Psychiatry.*

eFigure 4g - Forest plot - clinically relevant abnormality subtype: pituitary

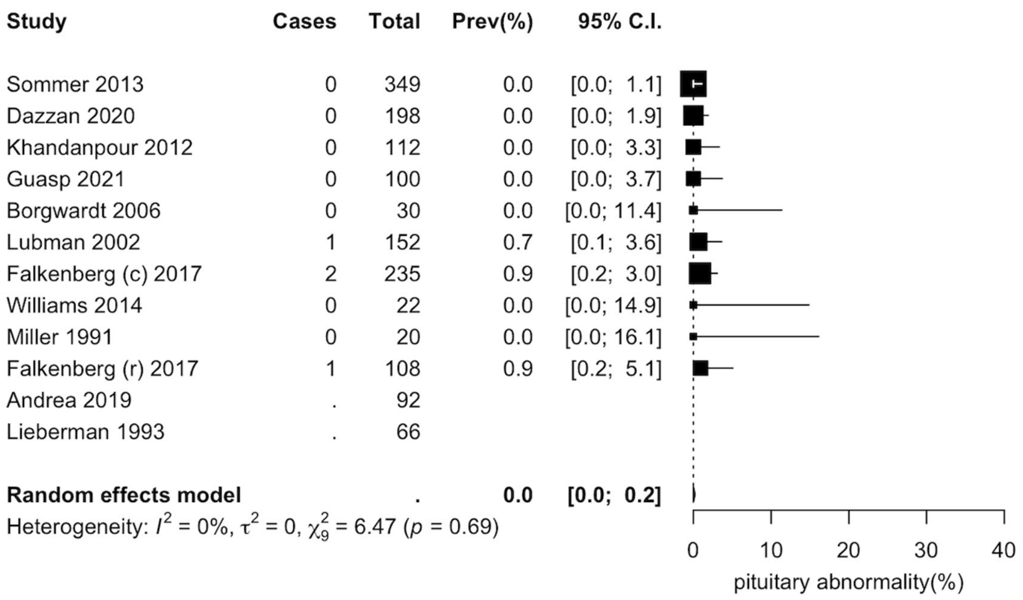

eFigure 4h - Forest plot - clinically relevant abnormality subtype: other

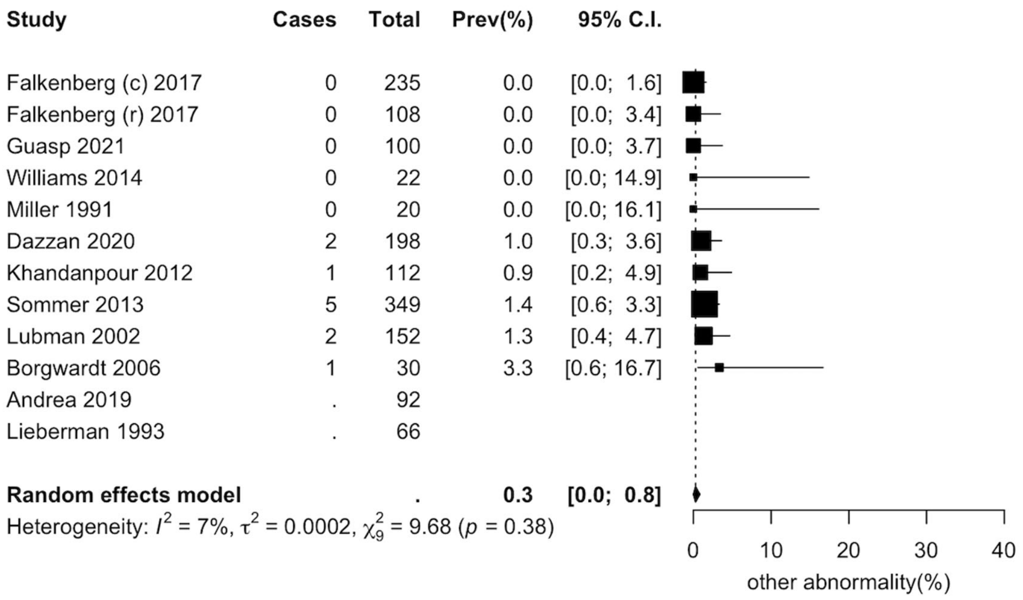

© 2023 Blackman G et al. *JAMA Psychiatry.*

eFigure section 5: Risk ratio of MRI abnormalities in FEP: Forest plots of all MRI abnormalities in psychosis

eFigure 5a: Forest plot: all abnormalities

Study	FEP Events	FEP Total	Controls Events	Controls Total	RR	95%-CI	Weight
Sommer 2013	85	349	192	722	0.92	[0.7; 1]	12.7%
Lubman 2002	34	152	23	98	0.95	[0.6; 2]	12.3%
Falkenberg (r) 2017	6	108	4	98	1.36	[0.4; 5]	9.6%
Dazzan 2020	96	198	44	115	1.27	[1.0; 2]	12.7%
Falkenberg (c) 2017	36	235	4	66	2.53	[0.9; 7]	10.6%
Borgwardt 2006	12	30	3	26	3.47	[1.1; 11]	10.0%
Lieberman 1993	20	66	2	42	6.36	[1.6; 26]	9.0%
Miller 1991	10	20	6	72	6.00	[2.5; 14]	11.0%
Khandanpour 2012	73	112	14	525	24.44	[14.3; 42]	12.1%
Andrea 2019	13	92	.	.			0.0%
Williams 2014	6	22	.	.			0.0%
Guasp 2021	4	100	.	.			0.0%
**Random effects model**		**1484**		**1764**	**2.76**	**[1.3; 6]**	**100.0%**

Heterogeneity: $I^2 = 95\%$, $\tau^2 = 1.1779$, $p < 0.01$

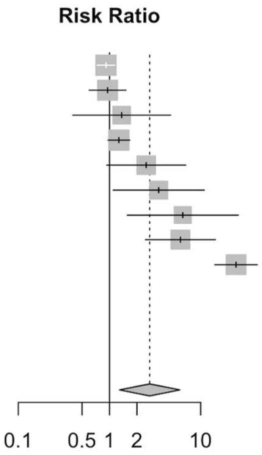

eFigure 5b: Forest plot: clinically relevant abnormalities

Study	FEP Events	FEP Total	Controls Events	Controls Total	RR	95%-CI	Weight
Sommer 2013	36	349	85	722	0.88	[0.6; 1]	42.2%
Falkenberg (c) 2017	22	235	4	66	1.54	[0.6; 4]	20.8%
Lubman 2002	13	152	5	98	1.68	[0.6; 5]	21.5%
Falkenberg (r) 2017	4	108	1	98	3.63	[0.4; 32]	6.9%
Dazzan 2020	5	198	0	115	6.40	[0.4; 115]	4.2%
Borgwardt 2006	7	30	0	26	13.03	[0.8; 218]	4.4%
Andrea 2019	3	92	.	.			0.0%
Khandanpour 2012	3	112	.	525			0.0%
Lieberman 1993	.	66	.	42			0.0%
Miller 1991	2	20	.	72			0.0%
Williams 2014	0	22	.	.			0.0%
Guasp 2021	.	100	.	.			0.0%
**Random effects model**		**1484**		**1764**	**1.53**	**[0.8; 3]**	**100.0%**

Heterogeneity: $I^2 = 38\%$, $\tau^2 = 0.1990$, $p = 0.15$

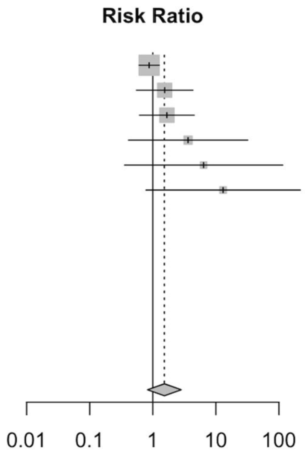

© 2023 Blackman G et al. *JAMA Psychiatry.*

eFigure 6: Forest plot of clinically relevant MRI abnormalities: Leave one out sensitivity analysis

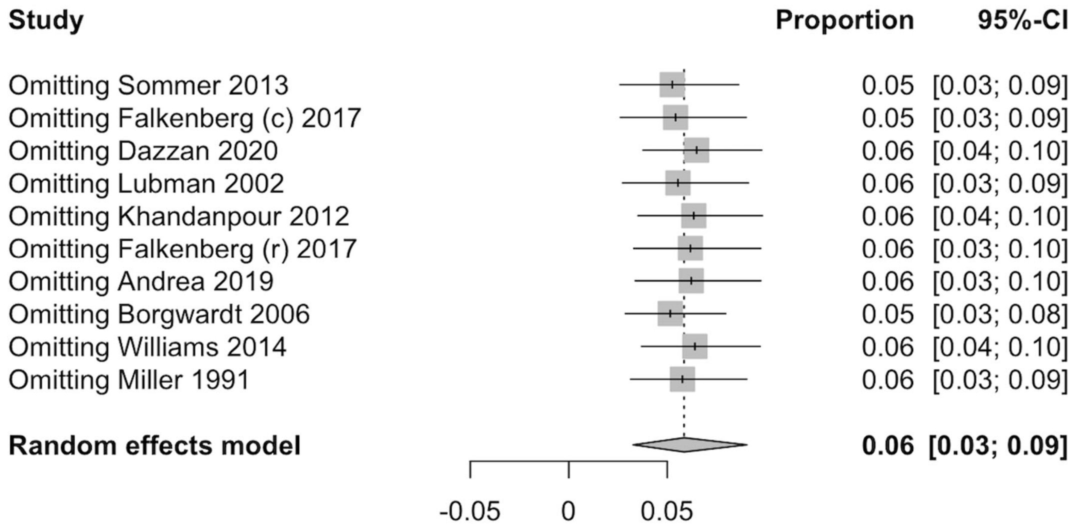

© 2023 Blackman G et al. *JAMA Psychiatry.*

eTable 1: Summary of included studies

Study	Continent	Recruitment	FEP (n)	HC (n)	Age (FEP)	FEP female (n)	HC female (n)	HC (age)	Psychosis Duration (weeks)	Field Strength (Tesla)	Quality Assessment	AP exposure (n)
Andrea et al (2019)	N. America	Clinical	92	-	20	nr	-	-	nr	nr	5	nr
Borgwardt et al (2006)	Europe	Clinical	30	26	30	8	9	23	nr	1.5	7	14
Dazzan et al (2020)	Europe	Research	198	113	25	53	42	25	17	3	4	nr
Falkenberg et al (c))2017)	Europe	Clinical	235	66	24	79	37	24	nr	3	5	195
Falkenberg et al (r) (2017)	Europe	Research	108	98	26	37	40	30	nr	1.5	8	77
Guasp et al (2021)	Europe	Research	100	-	30	44	-	-	4	nr	5	100
Khandanpour et al (2012)	Europe	Clinical	112	525	59	42	195	35	nr	1.5 / 3.0	4	0
Lieberman et al (1993)	N. America	Clinical	66	42	24	30	22	29	52	1	8	nr
Lubman et al (2002) [1]	Australia	Research	152	98	22	48	36	27	26	1.5	6	nr
Miller et al (1991)	N. America	Research	20	72	60	14	44	62	90	1.5	5	nr
Sommer et al (2013)	Europe	Research	349	722	nr	nr	315	34	nr	1.5	5	nr
Williams et al (2014)[1]	N. America	Clinical	22	-	21	nr	-	-	nr	nr	5	nr
Zanetti et al (2008)	S. America	Research	129	102	29	58	48	30	40	1.5	5	76

First Episode Psychosis (FEP), Healthy Control (HC), Anti Psychotic (AP), not reported (nr). Continuous variable rounded to nearest whole number. Dataset of Dazzan et al (2020) based upon personal correspondence. [1]sample age based upon mid point of reported range.

© 2023 Blackman G et al. *JAMA Psychiatry.*

eTable 2: Summary of recruitment, screening, and matching criteria of healthy controls

Study	Recruitment	Screening	Matching Criteria
Andrea et al 2019			
Borgwardt et al 2006	students of a trade school, hospital staff, and general advertisements	no history of psychiatric illness, head trauma, neurological illness, serious medical or surgical illness, substance abuse, or family history of psychiatric disorders (based on clinical interview)	not reported
Dazzan et al 2020	recruited across sites in Denmark (14), Spain (23), Ireland (19), Italy (14), Netherlands (9) and UK (34)	No history of psychiatric illness or MRI contraindications	not reported
Falkenberg et al (r) 2017	Random sample of population-based healthy comparison participants aged 16–64 years	Not reported	area of residence
Falkenberg et al (c) 2017	not reported	Not reported	not reported
Guasp et al 2021			
Khandanpour et al 2012	not reported	Not reported	not specifically matched. Compared to large sample of previously collected scans of 'normal volunteers' at the same imaging centre, reported in Hoggard et al (2008)
Lieberman et al 1993	from the community and from medical center staff through advertisement in the local media	No history of exclusionary medical or psychiatric illness (structured clinical interview; SCID) or history of drug abuse	age, gender, race, socioeconomic status, height
Lubman et al 2002	Ancillary hospital staff approached and general advertisements.	No personal or family history of psychiatric illness	socio-demographic area
Miller et al 1991	Advertisement in a local paper	No current or past psychotic, major affective, alcohol or substance abuse disorders, scored over 24 on the MMSE. Same exclusion criteria as patients.	age
Sommer et al 2013	newspaper advertisements and notice boards	No history of psychotic disorders or other mental health problems.	age, sex

© 2023 Blackman G et al. *JAMA Psychiatry.*

Williams et al 2014			
Zanetti et al 2008	next-door neighbours	No psychotic symptoms (Psychosis Screening Questionnaire; Structured Clinical Interview for DSM Disorders [SCID] for the assessment of other psychiatric disorders). Same exclusion criteria as psychosis group (screened for substance use with the Alcohol Use Disorders Identification Test (AUDIT) and the South Westminster Questionnaire; diagnoses of substance abuse or dependence were assessed using the SCID. Handedness was assessed with Annett's Hand Preference Questionnaire. Medical history, including data on cerebrovascular risk factors and information about antipsychotic drug treatment were obtained from case notes and interviews with individuals and/or family members.<br><br>Additional exclusion criteria were:<br>(a) history of head injury with loss of consciousness<br>(b) presence of neurological disorders or any organic disorders that could affect the central nervous system<br>(c) moderate or severe mental retardation<br>(d) contraindications for MRI scanning.	age (within 5 years) and gender

© 2023 Blackman G et al. *JAMA Psychiatry.*

eTable 3: Newcastle Ottawa Scale results

Study	Target population was a close representation of national population	The sampling frame was a true or close representation of target population	There was random selection to select the sample or a census was undertaken	Likelihood of response bias was minimal	Data was collected directly from the subject	There was an acceptable case definition	Reliability and validity of the study instrument that measured parameter of interest	Same model of data collection used for all subjects	Appropriate length of the shortest prevalence period for the parament of interest	Appropriate numerator(s) and denominator(s) for the parameter of interest	Total Score
Andrea et al (2019)	1	0	1	0	1	1	0	0	0	1	5
Bogwardt et al (2006)	1	1	1	0	1	1	1	0	0	1	7
Dazzan et al (2020)	1	0	0	0	1	1	0	0	1	0	4
Falkenberg et al (2017)[1]	1	1	1	1	1	1	0	1	0	1	8
Falkenberg et al (2017) [2]	1	0	1	1	0	0	0	1	0	1	5
Guasp et al (2021)	1	0	1	1	1	1	0	0	0	0	5
Khandanpour et al (2012)	0	0	0	1	0	1	0	1	0	1	4
Lieberman et al (1993)	1	1	1	1	1	1	1	1	0	0	8
Lubman et al (2002)	1	1	0	1	0	0	1	1	0	1	6
Miller et al (1991)	0	1	0	1	1	1	1	0	0	0	5
Sommer et al (2013)	1	0	1	1	0	0	1	1	0	0	5
Williams et al (2014)	1	1	0	0	1	1	0	0	1	0	5
Zanetti et al (2008)	1	1	0	0	1	1	1	0	0	0	5

[1]research subsample [2]clinical subsample

© 2023 Blackman G et al. *JAMA Psychiatry.*

eTable 4: Neuroanatomical Groupings

Ventricular	White Matter	Vascular	Atrophy	Cyst	Tumour	Pituitary	Other[1]	Excluded
Enlarged Ventricle	T2 Hyperintensity	Arteriovenous Malformation	Moderate Global Volume Loss	Arachnoid Cyst	Meningioma	Sella Partially Empty	Dolycocephaly	Sinus Abnormalities
Asymmetry of Ventricles	White Matter Hyperintensity	Brain Infarct	Severe Diffuse Volume Loss	Retroecrebellar Cyst	Cerebral Tumour	Sella Empty	Cortical Dyplasia	Abnormalities Of Ethmoidal Cells
Cavum Septi Pellucidi	Periventricular Hyperintensities	Lacunae	Localised Atrophy (e.g. Frontal, hypocampal)	Pineal Cyst	Space Occupying Lesion		HIV Encephalopathy	Adenoids
Absent Septum Pellucidum	Small Vessel Ischaemic Change	Brain Micro Bleed		Connatal Cyst	Harmatoma		Calficiation of Anterior Cerebral Falx	Mucous Retention Cyst
Cavum Vergae								
	Leukoencephalopathy	Post-Ischaemic Lesion		Focal Cystic Encephalomalacia			Mega Cisterna Magna	Concha Bullosa
	Demyelinating Disease	Cerebral Anneurysm		Pituitary cyst			Increased T2 attenuated inversion recovery change gliosis	Mastoid Abnormalities
	Periventricular Leukomalacia	Angioma		Choroidal Fissure Cyst				Mucosal Polyp
		Hamartoma						Hypo Ostosis Frontalis
		Subdural Effusion						Skull Abnormalities
		Aberrant Vessel Structure						Extra Cranial Abnormalities
		Enlarged Perivascular Spaces (Virchow–Robin Space)						Prominent Cerebellar Tonsils
		Cavernoma						
		Amyloid angiopathy						
		Cerebral haemorrhage						

[1]Defined as an abnormality not falling into any of the other predefined categories

© 2023 Blackman G et al. *JAMA Psychiatry.*

eReferences

Albon, E., Tsourapas, A., Frew, E., Davenport, C., Oyebode, F., Bayliss, S., Arvanitis, T., & Meads, C. (2008). Structural neuroimaging in psychosis: a systematic review and economic evaluation. *Health Technol Assess*, *12*(18), iii-iv, ix-163. https://doi.org/10.3310/hta12180

Forbes, M., Stefler, D., Velakoulis, D., Stuckey, S., Trudel, J. F., Eyre, H., Boyd, M., & Kisely, S. (2019). The clinical utility of structural neuroimaging in first-episode psychosis: A systematic review. *Aust N Z J Psychiatry*, *53*(11), 1093-1104. https://doi.org/10.1177/0004867419848035

Goulet, K., Deschamps, B., Evoy, F., & Trudel, J. F. (2009). Use of brain imaging (computed tomography and magnetic resonance imaging) in first-episode psychosis: review and retrospective study. *Can J Psychiatry*, *54*(7), 493-501. https://doi.org/10.1177/070674370905400711

Sommer, I. E., de Kort, G. A., Meijering, A. L., Dazzan, P., Hulshoff Pol, H. E., Kahn, R. S., & van Haren, N. E. (2013). How frequent are radiological abnormalities in patients with psychosis? A review of 1379 MRI scans. *Schizophr Bull*, *39*(4), 815-819. https://doi.org/10.1093/schbul/sbs037

© 2023 Blackman G et al. *JAMA Psychiatry.*
