# Supplementary material for: Prevalence of Neuroradiological Abnormalities in First-Episode Psychosis: A Systematic Review and Meta-analysis
Source: JAMA Psychiatry. 2023 Jul 12;80(10):1047–54. doi: 10.1001/jamapsychiatry.2023.2225 (PMC10339221; doi:10.1001/jamapsychiatry.2023.2225)
Supplement: Supplement 2. — Data sharing statement [file jamapsychiatry-e232225-s002.pdf]

Data Sharing Statement

Data
Data available: Yes
Data types: Deidentified participant data
How to access data: https://github.com/grahamblackman/FEP_MRI_anormality_Meta
When available: With publication

Supporting Documents
Document types: Statistical/analytic code
How to access documents: https://github.com/grahamblackman/FEP_MRI_anormality_Meta
When available: With publication

Additional Information
Who can access the data: researchers
Types of analyses: for research purposes
Mechanisms of data availability: data available open access
